# Changes in HSP gene and protein expression in natural scrapie with brain damage

**DOI:** 10.1186/1297-9716-42-13

**Published:** 2011-01-24

**Authors:** Carmen Serrano, Rosa Bolea, Jaber Lyahyai, Hicham Filali, Luis Varona, Ane Marcos-Carcavilla, Cristina Acín, Jorge H Calvo, Magdalena Serrano, Juan J Badiola, Pilar Zaragoza, Inmaculada Martín-Burriel

**Affiliations:** 1 Laboratorio de Genética Bioquímica (LAGENBIO), Facultad de Veterinaria, Universidad de Zaragoza, Miguel Servet 177, 50013 Zaragoza, Spain; 2 Centro de Investigación en Encefalopatías y Enfermedades Transmisibles Emergentes, Universidad de Zaragoza, Miguel Servet 177, 50013 Zaragoza, Spain; 3 Unidad de Genética Cuantitativa y Mejora Animal, Facultad de Veterinaria, Universidad de Zaragoza, Miguel Servet 177, 50013 Zaragoza, Spain; 4 Departamento de Mejora Genética Animal, INIA, Ctra La Coruña Km 7.5, 28040 Madrid, Spain; 5 Unidad de Tecnología en Producción Animal, CITA, Av. de Montañana, 930, 50059 Zaragoza, Spain

## Abstract

Heat shock proteins (Hsp) perform cytoprotective functions such as apoptosis regulation and inflammatory response control. These proteins can also be secreted to the extracellular medium, acting as inflammatory mediators, and their chaperone activity permits correct folding of proteins and avoids the aggregation of anomalous isoforms. Several studies have proposed the implication of Hsp in prion diseases. We analysed the gene expression and protein distribution of different members of the Hsp27, Hsp70, and Hsp90 families in the central nervous system of sheep naturally infected with scrapie. Different expression profiles were observed in the areas analysed. Whereas changes in transcript levels were not observed in the cerebellum or medulla oblongata, a significant decrease in *HSP27 *and *HSP90 *was detected in the prefrontal cortex. In contrast, *HSP73 *was over-expressed in diencephalons of scrapie animals. Western blotting did not reveal significant differences in Hsp90 and Hsp70 protein expression between scrapie and control animals. Expression rates identified by real-time RT-PCR and western blotting were compared with the extent of classical scrapie lesions using stepwise regression. Changes in Hsp gene and protein expression were associated with prion protein deposition, gliosis and spongiosis rather than with apoptosis. Finally, immunohistochemistry revealed intense Hsp70 and Hsp90 immunolabelling in Purkinje cells of scrapie sheep. In contrast, controls displayed little or no staining in these cells. The observed differences in gene expression and protein distribution suggest that the heat shock proteins analysed play a role in the natural form of the disease.

## Introduction

Transmissible spongiform encephalopathies (TSE) are fatal neurodegenerative diseases that include scrapie in sheep, bovine spongiform encephalopathy in cattle, and several human neuropathies such as Creutzfeldt-Jakob disease or fatal familial insomnia. These diseases are characterised by the accumulation in the central nervous system of an abnormally folded version (PrP^Sc^) of a normal cellular protein, PrP^C^. The related neurological lesions include spongiform changes in grey matter, intraneuronal vacuoles in particular nuclei of the brain stem, gliosis, and neuronal degeneration [1-5]. Although various mechanisms have been proposed to explain neuronal death in prion diseases, apoptosis and autophagy are the types of cell death considered most likely to be involved [6].

Our group has been investigating the molecular mechanisms underlying neuronal apoptosis in ovine naturally infected with scrapie. Despite the induction of Bax in scrapie brains, neurons suffering this type of Programmed Cell Death (PCD) were not observed in natural scrapie, suggesting either an extremely low number of cells undergoing apoptosis or the existence of neuroprotective mechanisms [7,8]. Expression profiles of other genes involved in different apoptotic pathways indicate that both pro and anti-apoptotic mechanisms are activated in prion-infected tissues [9]. Nevertheless, other factors could be involved in the regulation of cell death in natural scrapie.

It has been shown that over-expression of an individual heat shock protein (Hsp) can provide a protective effect against damaging stimuli in the same manner as can a mild Hsp-inducing stress [10,11]. For example, culture neurons derived from the central and peripheral nervous systems as well as different neuronal cell lines are protected against thermal or ischemic stress by over-expression of Hsp [12,13].

Hsp27, Hsp70, and Hsp90 (each named according to its mass in kilodaltons) are three of the best-characterised Hsp families. The anti-apoptotic role of these Hsp is mediated by different mechanisms. Hsp27 inhibits the activation of caspase-3, induced after cytochrome-c release from mitochondria and after the activation of death receptors such as Fas [14,15]. The other two chaperones (Hsp70 and Hsp90) have also been reported to target the mitochondrial pathway at different stages. Hsp90 binds to Apaf-1 and prevents its interaction with cytochrome-c [16], and Hsp70 prevents cytochrome-c/Apaf-1 complex formation by interacting with caspase-9 [17,18]. Hsp70 also antagonizes the caspase-independent mitochondrial pathway by binding directly to AIF [19] and inhibiting its nuclear translocation [20].

Detailed studies of individual Hsp also suggest that they play a role in ensuring the correct protein folding of other proteins within the cells, acting as so-called "molecular chaperones" [21]. Most neurodegenerative diseases are associated with events of protein misfolding. The accumulation of misfolded protein aggregates can overwhelm the ubiquitin-proteasomal system, inducing apoptosis and increasing neuronal vulnerability to subsequent insults [22]. Due to their anti-apoptotic and chaperone roles, Hsp are being considered for therapeutic use in neurodegenerative diseases such as Alzheimer, disease, Parkinson disease and stroke (reviewed in [23]). In addition to the functions mentioned, potential involvement of Hsp in the conversion of prion protein to its protease-resistant form and propagation of the infectious protein is being investigated (reviewed by [24]).

Analysis of the expression patterns of genes and proteins is a good tool to identify the possible implication of these proteins in TSE. Comparison of gene expression profiles with the distribution of scrapie related lesions in the brain could reveal the role of different factors in the neuropathology of prion diseases. A recent report from our group identified the association between activation of the mitochondrial apoptosis pathway and early stages of PrP^Sc ^deposition, whereas induction of the extrinsic pathway was related to reactive gliosis and neuronal loss [9]. During prion infection, gene and protein expression, principally of Hsp70 family members, is increased in different prion disease models [25-29].

At present, little is known about the expression and distribution of Hsp in a model of natural infection such as naturally scrapie-infected sheep. Only recently, *HSP90AA1 *has been analysed as a possible candidate gene involved in scrapie susceptibility in sheep [30], probably by modulating the incubation period of this disease [31]. We present here the first gene expression analysis of four members of the *HSP27 *(*HSPB1*), *HSP70 *(the constitutive form *HSP72/HSPA1A *and the inducible *HSP73/HSPA8*), and *HSP90 *(the inducible form, *HSP90AA1*) gene families, their protein expression profiles, and their distribution in the central nervous systems of sheep naturally infected with clinical-stage scrapie. The association between gene and protein expression data and scrapie histopathological lesions has also been investigated. These heat shock proteins have been chosen for their known roles as chaperones and apoptosis modulators, as well as their possible functions in prion diseases.

## Materials and methods

### Animals and sample collection

Thirteen females of the *Rasa Aragonesa *sheep breed were included in a recent study that focused on the analysis of the molecular mechanism of apoptosis in scrapie [9]. The same RNA and tissue samples were used in the present work. Briefly, scrapie was diagnosed in vivo in eight of these sheep [32] and confirmed after animals were sacrificed [33]. When these animals were sacrificed, all exhibited clinical signs of terminal-stage scrapie. The five control animals were selected from a different flock of the same breed, in which no scrapie cases have been reported to date. All animals analysed were 3 to 6 years old and displayed the ARQ/ARQ genotype for the *PRNP *gene, which is the most susceptible genotype in this ovine breed. Care and handling of the animals were performed according to the rules established by the National Research Council.

Immediately after sacrifice, samples from the medulla oblongata, diencephalon, cerebellum, and prefrontal cortex were harvested from each sheep. The bilateral nature of scrapie lesions calls for half-trimming of the brain. One half was placed in RNA*later*^® ^(Ambion, Austin, TX, USA) for 24 h at 4°C and then frozen at -80°C until RNA extraction; the other half was placed in formalin-fixative and paraffin-embedded for further histopathological analysis. Sections adjacent to the sampled areas were frozen in liquid nitrogen and stored at -80°C for protein extraction. The FASTH system (Prionics AG, Zurich, Switzerland) was used to homogenise the tissue samples collected for RNA and protein extraction.

### Gene expression

Total RNA was isolated from tissue samples using the RNeasy Lipid Mini Kit (Qiagen, Hilden, Germany) as previously described [9]. Complementary DNA for each animal and central nervous system (CNS) region was synthesised from 2 μg of total RNA using random hexamers and the Superscript First Strand Synthesis System for RT-PCR (Invitrogen, Carlsbad, CA, USA). The expression rates of four genes involved in the heat shock response were evaluated using quantitative real-time RT-PCR. Primer express 2.0 software (PE Applied Biosystems, Foster City, CA, USA) was used to design primers and probes based on known bovine sequences for *HSP72 *(*HSPA1A*), *HSP73 *(*HSPA8*), and *HSP27 *(*HSPB1*) (Table 1). The primers corresponding to the *HSP90 *(*HSP90AA1*) gene were previously described in ovine species [30]. The identity of the PCR products was confirmed by sequencing and BLAST comparison with the GenBank database. The length of the RT-PCR products is shown in Table 1.

**Table 1:** Genes analysed and real time RT-PCR conditions

Genes	GenBank^1^	Species^2^	Primers (5'→ 3')^3^	bp^4^	PCR conditions^5^			
					**Ta**	**[nM]**	**r^2^**	**Slope**

*HSP27*	BT021550	Bovine	F: TGGCGCGTGTCCCTGGA	80	60	300	0.990	-3.33
			R: GTGATCTCCACCACGCC			300		
*HSP72*	AY662497	Bovine	F: ACCCGCAGAACACGGTGTT	119	60	900	0.995	-3.33
			R: AGGCTTGTCTCCGTCGTTGA			900		
*HSP73*	NM_174345	Bovine	F: CAACCTGCTTGGCAAGTTTGA	108	60	900	0.990	-3.20
			R: GAAACATTGAGGATGCCATTGG			900		
*HSP90*	[30]	Ovine	F: AGTCTGGAGGATCCCCAGACA	78	60	300	0.994	-3.32
			R: GGGTCATCCTCGTCAATACCA			300		

^1 ^GenBank accession numbers of the sequences used for primer design.

^2 ^Species of origin of the sequences.

^3 ^Primers (F: Forward and R: Reverse) used for the gene amplification.

^4 ^Length of the amplicon in base pairs (bp).

^5 ^Real-time RT-PCR conditions for gene expression analyses: annealing temperature (Ta), primer concentration ([nM]), correlation coefficient (r^2^) and slope of the standard curve.

Gene expression was analysed using SYBR^® ^Green (PE Applied Biosystems) assays. A dissociation curve protocol was run after every real-time RT-PCR reaction in order to identify the presence of spurious PCR bands or high levels of primer dimers. The appropriate primers used for amplification of all genes, as well as their concentrations, are shown in Table 1.

Real-time RT-PCR amplifications were performed in an ABI-Prism 7000 Sequence Detection System (PE Applied Biosystems). All real-time RT-PCR reactions were run in triplicate in a total reaction volume of 10 μL using 10-20 ng of cDNA as template. Amplification of cDNA by PCR was achieved using universal cycling conditions with an initial 10 min activation and denaturation step at 95°C, followed by 40 cycles of 15 s at 95°C and 30 s at the suitable annealing temperature (Table 1). The levels of gene expression were determined using the comparative Ct method. A normalisation factor (NF) was used to determine the expression level of each gene in each sample as described [7,34].

### Protein extraction and Western blot analysis

At least 0.2 g of the brain frozen sections were homogenised in 2 mL of Prionics^® ^Check Western homogenisation buffer (Prionics AG, Zurich, Switzerland) and centrifuged twice at 10 000 × *g *for 10 min at 4°C. Supernatants containing total protein extracts were recovered and protein concentrations measured by BCA (bicinchoninic acid) protein assay (Sigma-Aldrich, St. Louis, MO, USA). After denaturation at 95°C for 5 min, protein extracts (50 μg of total protein) were subjected to SDS/PAGE (8% polyacrylamide) at 120 V for 1 h 30 min and transferred to PVDF membranes (GE Healthcare, Little Chalfont Buckinghamshire, UK) at 100 V for 2 h using a Mini-PROTEAN 3 system (Bio-Rad, Hercules, CA, USA).

The PVDF membranes were treated with blocking solution (TBS buffer, 0.5% Tween 20 and 5% non-fat milk) at 4°C overnight, then incubated for 1 h with the appropriate primary antibody diluted in blocking buffer (1:1000 for mouse monoclonal anti-Hsp70, Santa Cruz Biotechnology, Santa Cruz, CA, USA; 1:1000 for mouse monoclonal anti-Hsp90, Stressgen, Ann Arbor, MI, USA; 1:500 for mouse monoclonal anti-Hsp27, Santa Cruz Biotechnology). Antibodies against Hsp70 and Hsp90 react with both the constitutive (Hsp72 and Hsp90β, respectively) and the inducible (Hsp73 and Hsp90α, respectively) forms of these proteins. Anti-GAPDH antibody (rabbit polyclonal IgG; Santa Cruz Biotechnology) diluted 1:4000 in blocking buffer was used for normalisation of the protein expression values. Next, the membranes were incubated for 1 h with HRP-conjugated secondary antibody diluted 1:4000 in blocking buffer (goat anti-mouse IgG-HRP for anti-Hsp or goat anti-rabbit IgG-HRP for anti-GAPDH; Santa Cruz Biotechnology). Three washes with TBS-0.5% Tween 20 were performed between incubation periods. Western blots were developed using the ECL Plus Western Blotting system (GE Healthcare, Little Chalfont Buckinghamshire, UK) and exposed to X-ray films. The antibody used for Hsp27 immunodetection did not reveal any specific band in the CNS protein extracts under the conditions employed in this work. Therefore, the quantification analysis was performed based on the Hsp70 and Hsp90 bands, using the spot density data calculated with the AlphaEase FC software (Alpha Innotech, San Leandro, CA, USA).

Additionally, in order to check the linearity of the band densities obtained by the X-ray methodology, we performed a Western blot assay with a dilution series (12.5 μg, 25 μg, 50 μg, 75 μg and 100 μg) of a protein extract of CNS from a negative sheep.

### Immunohistochemical detection of Hsp

Five-micrometer-thick sections from the medulla oblongata (MO), diencephalon (D), cerebellum (C), and prefrontal cortex (PFC) from scrapie-infected and control sheep were processed for immunochemical detection of Hsp27, Hsp70, and Hsp90 proteins with the antibodies used for Western blot. The unmasking protocol used included a steam heat procedure. In brief, rehydrated slices were immersed in preheated antigen retrieval solution (citrate buffer 1×, DAKO), exposed to heat in a pressure cooker for 10 min, and cooled slowly for 20 min. After treatment, the sections were incubated with blocking reagent (DAKO, Glostrup, Denmark) for 10 min to block endogenous peroxidase activity. Next, sections were incubated for 1 h at room temperature (RT) with the primary antibodies used for western blotting (see above): Hsp70 (1:150), Hsp90 (1:300), and Hsp27 (1:50, 1:100, and 1:150). The enzyme-conjugated polymer Envision (DAKO; 30 min) was used as the visualisation system and DAB (DAKO; 10 min) was used as the chromogen. Sections were counterstained by treatment with haematoxylin.

The reactivity of antibodies was confirmed in sheep breast carcinoma tissue (positive controls for Hsp70 and Hsp90) and in normal breast tissue (positive control for Hsp27). The specificity of immunoreactivity was demonstrated by Western blot and by the elimination of labelling in all cases when primary antibodies were omitted (see above). In routine immunoreactions, the omission of primary antibodies served as a negative control, whereas breast carcinoma lymph node tissue sections were included as positive controls.

### Histopathology, prion, and caspase-3 detection

Histopathological study of the medulla oblongata, diencephalon, cerebellum, and prefrontal cortex has been previously reported [9]. Neuronal vacuolation and neuropil spongiosis were evaluated in hematoxylin-eosin-stained slices and PrP^Sc ^detection was performed for adjacent sections, as previously described [35]. Astrogliosis was evaluated on the basis of glial fibrillary acidic protein (GFAP) immunoreactivity, and induction of caspase-dependent apoptosis was evaluated by immunohistochemical detection of the active form of caspase-3. To visualise microglial cells, affinity histochemistry was applied using biotinylated Lectin I (Isolectin B4) from *Bandeiraea *(*Griffonia*) *simplicifolia *(LGS) (Vector Laboratories, Peterborough, UK), following the previously described protocol [36]. Briefly, dewaxed sections with an initial block of endogenous peroxidase activity were incubated overnight with this lectin. The washing buffer was supplemented with CaCl_2_, MgCl_2_·6H_2_O, and MnCl_2_·4H_2_O (1 mM). Binding was visualised with the avidin-biotin complex (ABC Complex; Thermo Scientific Pierce Labs, Rockford, IL, USA) and 3, 3'-diaminobenzidine.

Global quantification of scrapie lesions (spongiosis and neuronal vacuolation), PrP^Sc^, GFAP, and caspase-3 immunoreactivity, as well as lectin staining, were scored on a scale ranging from 0 to 5. The subjective scores were determined by two observers.

### Statistical analysis

Quantitative results obtained from real-time RT-PCR assays were expressed as mean ± standard error of the mean (SEM) values. Gene expression means for the control group were arbitrarily standardised to 1.0, and data for the scrapie-infected group were compared based on this calibrator. The Student's *t*-test analysis was applied to determine whether the differences observed between groups were statistically significant (*p *< 0.05). Differences in normalised data obtained from Western blot were also evaluated using the Student's *t*-test.

We studied the relationship between the gene and protein expression profiles of each chaperone and histopathological lesions by correlation. Further, in order to determine joint effects from several lesions we performed a stepwise regression with probabilities for forward selection and backward elimination set at *p *< 0.05. The model used for analysis of each combination of gene/protein and tissue lesion was

$$y_{i}=\mu +b_{1}pr_{i}+b_{2}nv_{i}+b_{3}sp_{i}+b_{4}as_{i}+b_{5}mi_{i}+b_{6}cp_{i}+e_{i}$$

where y_i _is the gene or protein expression profile of the i*th *individual; pr_i_, nv_i_, sp_i_, as_i_, mi_i _and cp_i _represent numerical scores of the i*th *individual for PrP^Sc^, neuronal vacuolisation, spongiosis, astrogliosis, microgliosis, and activation of caspase-3, respectively; *b_1_*, *b_2_*, *b_3_*, *b_4_,b_5 _*and *b_6 _*are the slopes of the multiple regression associated with each variable; and e_i _is the residual.

Finally, to confirm the differences in Hsp70 and Hsp90 immunolabelling in Purkinje cells, stained and non-stained Purkinje cells were counted in five microscope areas (20× magnification) for each animal. Percentages were converted to arcsin values before applying the Student's *t*-test.

Statistical analyses were performed using either SPSS (Chicago, IL, USA) or STATISTIX (Analytical Software, Tallahassee, FL, USA).

## Results

### *HSP *gene expression

Untreated samples and samples treated with reverse transcriptase showed differences of more than 6 cycles for every gene, indicating that genomic DNA was successfully removed (data not shown). Standard curves for all factors analysed displayed appropriate slopes and correlation values (Table 1).

**Figure 1:**
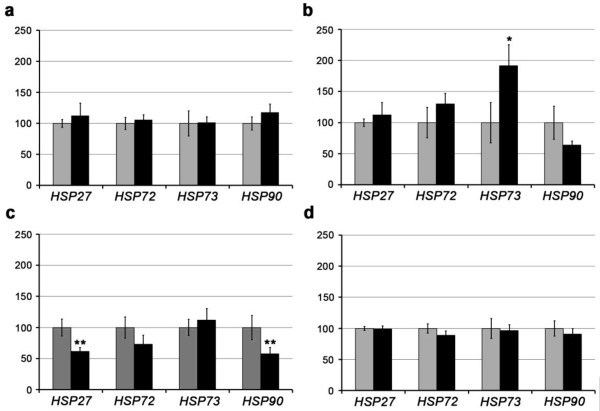
**Gene expression profiles of four chaperone genes (*HSP27*, *HSP72*, *HSP73*, and *HSP90*) in four CNS areas of control (grey bars) and scrapie (black bars) sheep in the medulla oblongata (a), diencephalon (b), prefrontal cortex (c), and cerebellum (d)**. Differences between groups were analysed using the Student's *t*-test (*p < 0.05, **p < 0.01).

The expression of the four *HSP *genes was analysed in four different areas of the central nervous system of scrapie-infected and control sheep. As Figure 1 shows, different gene expression profiles were observed in the analysed areas. Whereas changes in transcript levels were not observed in the cerebellum and medulla oblongata, significant decreases in *HSP27 *(*p *< 0.05) and *HSP90 *(*p *< 0.01) were detected in the prefrontal cortices of scrapie animals. In contrast, *HSP73 *was significantly over-expressed (*p *< 0.05) in infected diencephalons.

### Expression of Hsp

Tissue extracts from the medulla oblongata, diencephalon, cerebellum, and prefrontal cortex were subjected to Western blot analysis to examine quantitative changes in the expression of Hsp70 and Hsp90 in scrapie animals.

**Figure 2:**
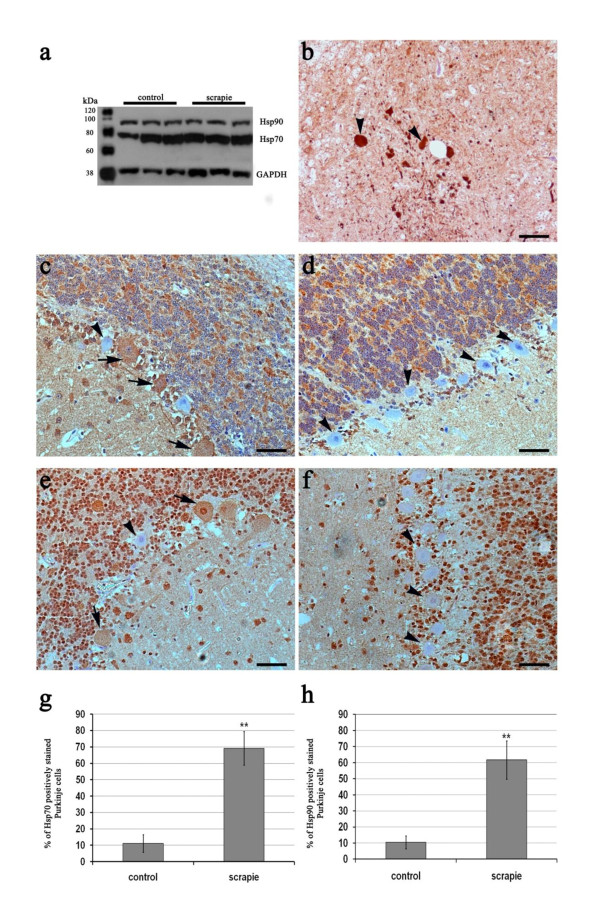
**Hsp protein expression and distribution in scrapie tissues.** Specificity of anti-Hsp90 and anti-Hsp70 antibodies in the ovine medulla oblongata, as detected by western blotting (a). Arrowheads indicate intense Hsp70 immunostaining of spheroids observed in medulla oblongata (b). Immunohistochemical determination of Hsp70 in scrapie (c) and control (d) animals; Hsp70-positive and -negative Purkinje cells are indicated with arrows and arrowheads, respectively. Immunohistochemical determination of Hsp90 in scrapie (e) and control (f) animals; Hsp90-negative Purkinje cells are indicated by arrowheads, and Hsp90-positive cells are indicated by arrows. Significant increase in the percentage of Purkinje cells staining positively for Hsp70 (g) and Hsp90 (h) in scrapie cerebella. ** *p *< 0.01 (Student's *t*-test).

A single band of ~70 kDa, approximately, was obtained by Western blotting, demonstrating the specificity of the anti-Hsp70 antibody (Figure 2). No statistically significant changes were observed between controls and scrapie-affected individuals with respect to global Hsp70 expression in the four CNS zones. Using the anti-Hsp90 antibody, Western blot also revealed a single band that corresponded to the expected value (~90 kDa) (Figure 2). As for Hsp70, no significant differences in Hsp90 expression were detected between controls and scrapie-affected animals in any area of the CNS.

The dilution curve assay showed adequate determination coefficients (r^2^) that indicate a linear correlation between the amount of loaded protein and band density. The r^2 ^values obtained for Hsp70, Hsp90 and GAPDH were 0.967, 0.964 and 0.966, respectively.

### Analysis of scrapie-related lesions

The neuropathological features of scrapie were evaluated in the four CNS areas of 5 control and 8 scrapie-infected sheep. These results have recently been published in the framework of an investigation of apoptosis in natural scrapie [9]. Lesion scores for each individual are presented as Additional file 1. Briefly, neuronal vacuolisation was detected only in affected animals, and the highest lesion levels were observed in the medulla oblongata and diencephalon. Neuropil spongiosis was observed in both groups of animals; however, the increased rate of this lesion in scrapie animals was statistically significant in all areas except the cerebellum. Prion protein deposition intensity varied by area; the origin of this difference was the low level of PrP^Sc ^immunolabelling observed in the prefrontal cortex.

Astrogliosis was evaluated by GFAP detection. A generalised increase in the astroglial marker GFAP was noticed in the scrapie-affected cases. Apparent hyperplasia and hypertrophy of stellate-shaped astroglial cells were observed in both the grey and white matter when compared to control brains, indicative of reactive astrogliosis. Differences between areas were not observed in either control or infected sheep.

Histochemical labelling with LGS revealed an increase in the number of activated microglial cells in the four analysed areas in infected animals. In both control and scrapie-affected animals, there was diffuse immunolabelling of the neuropil in the molecular layer of the cerebellar grey matter, as described by Vidal et al. [36]. Similar labelling of the perineuronal area of the cerebellar nuclei was observed. The morphology of the immunolabelled microglial cells was either ramified with an elongated rod-shaped nucleus or amoeboid with a more rounded nucleus, indicating activation.

Finally, cell death was also analysed by immunodetection of caspase-3, using an antibody that preferentially reacts with the activated form of this protein. In general, very weak staining was observed in the different CNS sections; the intensity and distribution of staining did not differ significantly between the scrapie and control groups.

### Relationship between gene and protein expression profiles and histopathological lesions

Single correlations were performed between every lesion and the gene and protein expression in the four analysed tissues. Significant negative correlations were detected between prion protein deposition and the expression of *HSP27 *(-0.677, *p *= 0.011), *HSP72 *(-0.672, *p *= 0.023) and *HSP90 *(-0.582, *p *= 0.047) genes; between spongiosis and *HSP27 *gene (-0.592, *p *= 0.033) and Hsp90 protein (-0.614, *p *= 0.034) expression and between vacuolation and Hsp90 protein expression (-0.694, *p *= 0.012) in the prefrontal cortex. Further a negative correlation was also detected between *HSP27 *gene expression and Caspase3 (-0.728, *p *= 0.017) in the cerebellum.

Stepwise regression was carried out to determine possible association between gene or protein expression profiles and joint effects of prion-related lesions in the four areas analysed. Table 2 shows the significant regression values obtained. Increases on *HSP *gene expression in the prefrontal cortex (*HSP27*, *HSP72 *and *HSP73*) and in the cerebellum (*HSP27*, *HSP73 *and *HSP90*) were associated with decreases of PrP^Sc ^(negative regression) and increases of GFAP (positive regression) immunolabelling. Inversed associations were observed in the medulla oblongata, but only for *HSP27*.

**Table 2:** Significant regression values between gene or protein expression and scrapie-related lesions, as obtained by stepwise regression analysis

	Prion	Spongiosis	Vacuolation	GFAP	LGS	Caspase3
**Medulla oblongata**						
*HSP27*	0.399 (0.003)^1^	-0.381 (0.016)	ns^2^	-0.443 (0.043)	ns	ns
*HSP72*	ns	ns	ns	ns	ns	ns
*HSP73*	ns	ns	ns	ns	ns	ns
*HSP90*	ns	ns	ns	ns	ns	ns
Hsp70p	ns		ns	ns	ns	ns
Hsp90p	ns	ns	ns	ns	ns	ns
**Cerebellum**						
*HSP27*	-0.178 (0.032)	ns	ns	0.294 (0.0430)	ns	ns
*HSP72*	ns	ns	ns	ns	ns	ns
*HSP73*	-0.719 (0.015)	0.317 (0.023)	ns	0.783 (0.034)	0.555 (0.018)	ns
*HSP90*	-0.465 (0.008)	0.182 (0.039)	ns	0.712 (0.01)	ns	ns
Hsp70p	ns	-0.014 (0.010)	ns	ns	0.022 (0.02)	0.026 (0.010)
Hsp90p	ns	ns	ns	ns	ns	ns
**Prefrontal cortex**						
*HSP27*	-0.228 (0.004)	ns	ns	0.267 (0.025)	-0.129 (0.038)	ns
*HSP72*	-0.347 (0.003)	ns	ns	0.277 (0.034)	ns	ns
*HSP73*	-0.271 (0.036)	ns	ns	0.381 (0.03)	ns	ns
*HSP90*	ns	ns	ns	ns	ns	ns
Hsp70p	ns	ns	-0.049 (0.015)	0.045 (0.013)	-0.023 (0.018)	ns
Hsp90p	ns	ns	-0.080 (0.002)	0.046 (0.015)	-0.024 (0.020)	ns
**Diencephalon**						
*HSP27*	ns	ns	ns	ns	ns	ns
*HSP72*	ns	ns	ns	ns	ns	ns
*HSP73*	ns	ns	ns	ns	ns	ns
*HSP90*	ns	ns	ns	ns	ns	ns
Hsp70p	ns	-0.805 (0.001)	ns	0.778 (0.002)	ns	ns
Hsp90p	ns	ns	ns	ns	ns	ns

^1^Significant *p*-values (*p *< 0.05) are shown in brackets.

^2 ^ns: regression values not statistically significant.

Microglia activation (LGS) was positively associated with *HSP73 *gene and Hsp70 protein expression in the cerebellum and negatively with *HSP27 *gene and Hsp70 and Hsp90 protein expression in the prefrontal cortex.

High *HSP73 *and *HSP90 *gene expression rates showed association with an increase of spongiosis in the cerebellum. However, low degrees of spongiosis were associated to higher *HSP27 *gene expression rates in the medulla oblongata and higher Hsp70 protein expression levels in the cerebellum and diencephalon. Increases on vacuolisation were associated with slight decreases of Hsp70 and Hsp90 protein expression in the prefrontal cortex. Finally, slight increases on Hsp70 protein expression were associated with the appearance of active caspase-3 immunoreactivity.

### Protein distribution

Cellular localisation of Hsp70 and Hsp90 was revealed by immunohistochemistry. Anti-Hsp27 antibody did not allow the immunohistochemical detection of ovine Hsp27 under the conditions employed here.

For Hsp70, immunohistochemistry images of the analysed areas showed a diffuse protein distribution with stained neuronal bodies, mild labelling of the neuropile, and granular labelling of glial cell cytoplasmic processes (Figure 2). No marked differences in staining intensity were detected between tissues samples from control and infected sheep. High variability within groups was observed. However, Hsp70 was over-expressed in Purkinje cells of most of the scrapie-infected animals (Figure 2). Moreover, in one infected sheep showing a large spongiform change, the expression of Hsp70 was low for all areas studied. In addition, positively labelled "spheroids" were noted in the brains of some affected sheep (Figure 2), most likely corresponding to foci of neuronal degeneration [36,37].

The antibody used for the detection of Hsp90 resulted in specific cytoplasmic staining in the carcinoma mammary gland sections (positive controls) as well as in CNS sections of the animals tested. The negative controls did not show any immunohistochemical reaction. High variability in Hsp90 immunoreactivity was observed in the medulla oblongata in scrapie and control animals. Intense staining was observed in either the neuronal bodies or neuropile, depending on the animal studied. Both patterns were also detected in the prefrontal cortex and diencephalon, without significant differences between animals (data not shown). Finally, the number of Hsp90-positive Purkinje cells was significantly higher in scrapie-affected animals' cerebella (*p *< 0.01) (Figure 2).

## Discussion

Neurodegeneration and gliosis are the main neuropathological features of prion diseases. However, the molecular mechanisms involved in these processes remain unclear. Previous research from our group showed the relationship between altered expression of genes involved in the regulation of apoptosis and scrapie-related lesions [7-9]; however, other factors may participate in scrapie neuropathology.

Formation of protein aggregates in neurons or in the extracellular matrix is an important characteristic in many neurodegenerative processes such as prion diseases, Alzheimer, Parkinson, or Huntington disease. It seems to be evident that the cellular machinery responsible for avoiding anomalous protein accumulation would be implicated in these pathologies. Therefore, chaperones could play an important role in neuronal degeneration [38]. In this context, little is known about natural models of TSE. The aim of this study was to analyse the gene and protein expression of four Hsp chaperones in the CNS of sheep naturally infected with scrapie; the chaperones analysed were chosen according to their known roles in apoptosis modulation or their possible functions in prion diseases.

Over-expression of *HSP70 *family genes has been reported in brain samples from scrapie-infected mice [25,27]. Our expression analysis revealed significant over-expression of *HSP73 *transcripts in scrapie diencephalons. Hsp70 prevents the accumulation and/or promotes the degradation of specific PrP conformers in fly models and protects against PrP neurotoxicity through unknown mechanisms [39]. In our study, *HSP73 *expression in diencephalons was independent of PrP^Sc ^immunolabelling (Table 2) suggesting an alternative role for this chaperone. We reported a lack of apoptosis induction despite the significant increase of *BAX *[8] and *BAK *[9] in scrapie diencephalons. Joining these results, we hypothesize that *HSP73 *could counteract the induction of apoptosis by these mitochondrial factors.

In contrast, in the prefrontal cortex, the expression of *HSP90 *and *HSP27 *genes decreased in the scrapie group. This *HSP90 *pattern is not in agreement with the Hsp90 up-regulation observed in murine models of Bovine Spongiform Encephalopathy (BSE) [28]. It is possible that mRNA levels do not correspond to protein expression. It has been suggested that under inflammation or ischemic processes, Hsp synthesis results from a post-transcriptional regulatory mechanism that involves mRNA stabilisation [40]. This is why we analysed the expression of Hsp proteins by Western blot. Interestingly, no significant differences were detected between controls and scrapie animals. An association analysis established differential distribution of certain polymorphisms in the 5' and 3' regions of the ovine *HSP90*AA1 gene in scrapie-susceptible animals [30]. Previously, other authors observed an important role of the 3'UTR region in post-transcriptional regulation of Hsp70 upon heat shock [41,42]. Thus, the decrease of the *HSP90 *gene expression observed in the prefrontal cortex could be caused by variation at transcriptional or post-transcriptional levels. Perhaps these mutations do not affect *HSP90 *translation, or, alternately, Western blot technique is not sensitive enough to detect certain changes in protein expression.

In addition, interindividual variability has been reported for natural scrapie [7-9,43]. This may mask small differences in gene and protein expression between healthy and scrapie-affected animals. For this reason, we performed an additional statistical analysis to detect whether a relationship exists between the expression pattern and the histopathological changes observed in scrapie animals. This methodology allows the analysis of the molecular mechanisms underlying these processes in the natural form of the disease. Although we are aware that the histopathological valuation does not correspond to a real scale, our procedure can reliably detect the association among different parameters. However, the lack of statistical power due to the limited number of individuals analysed could prevent the detection of additional associations.

Although stepwise regression results were different in the four areas analysed, it is worth mentioning that we identified a negative association between prion protein deposition and the expression of the *HSP *genes in the cerebellum (*HSP27*, *HSP73 *and *HSP90*) and prefrontal cortex (*HSP27, HSP72 *and *HSP73*). It has been demonstrated that Hsp70 members avoid protein aggregation, which leads to neurodegenerative diseases [44-46]. Thus, down-regulation of chaperones could contribute to the accumulation of anomalous proteins and finally to cell stress. A decrease of Hsp27 has been described in early stages of in vitro models of spinocerebellar ataxia type 3 [47] and type 7 [48], which are neurodegenerative disorders associated with an accumulation of polyglutamine-containing proteins.

Glial reaction is the other classical sign of natural scrapie. We previously reported a positive association between astrogliosis and the extrinsic pathway of apoptosis in the medulla oblongata and prefrontal cortex. In addition to apoptosis regulation, factors involved in this pathway can participate in inflammation [49], reactive gliosis and neuroinflammation [50,51]. Hsp are also secreted as inflammatory mediators that bind surface receptors and induce the production of nitric oxide, cytokines, and other immunoregulatory molecules. Levels of the small heat shock protein Hsp25 increased during reactive astrogliosis in a murine model of BSE [52], and Hsp70 expression has been reported in reactive astrocytes following ischemia [53,54]. Microglial activation by Hsp proteins, such as Hsp70 and Hsp90, results in clearance of amyloid peptide aggregates [55,56]. In agreement with this, in the present work GFAP immunolabelling was positively associated with the expression of several *HSP *genes and proteins in the areas where these genes were negatively associated with prion protein immunolabelling (cerebellum and prefrontal cortex). Then, high levels of *HSP *gene or protein expression would be associated to the prevention or degradation of prion protein aggregates and the presence of reactive astrocytosis in natural scrapie. Although a strong positive association was found between *HSP73 *gene expression and microglia staining in the cerebellum, our association results are not conclusive with respect to the role of *HSP *in microglia activation.

The Hsp analysed in this work were selected due to their known role in apoptosis regulation. In a previous study, searching for possible evidence of cell death mechanisms in natural scrapie by immunodetection of activated caspase-3 [9], we observed weak staining in all CNS sections without finding significant differences between control and scrapie animals. However, a positive association was found between caspase-3 and the mitochondrial pathway of apoptosis [9]. Since Hsp work as anti-apoptotic factors [15,17,19] we analysed the relationship between caspase-3 expression and Hsp gene and protein expression. Only Hsp70 protein expression in the cerebellum showed a positive correlation with caspase-3. The absence of an association in the remaining areas may reflect that, in general, Hsp are not involved in the regulation of neuronal death during natural disease, as was observed in the acute phase of epilepsy [57].

Finally, when cases of natural disease are studied, changes detected in certain cell populations are frequently not correlated with variations in gene or protein expression in the final tissue mixture, as we observed for Bax protein determination in the CNS of scrapie sheep [8]. For this reason, in order to confirm the previous results and elucidate the cell type involved in possible expression variability, an immunohistochemical assay of Hsp was performed in each area of the CNS. In general, diffuse and highly variable staining for Hsp70 and Hsp90 was observed in the four CNS areas of both groups of animals. Nevertheless, the over-expression of Hsp in Purkinje cells of scrapie cerebella was remarkable. Intense staining for Hsp72 in this cell type was also observed in Creutzfeldt-Jakob disease (CJD) patients [58]; accumulation of this protein in Purkinje cells supports the proposed neuroprotective role of Hsp in the natural form of the disease. Similarly, although caspase-3 immunostaining was present in cerebellar granular cells, we did not observe this staining in Purkinje cells, in either scrapie-affected or control sheep [8,9]. To our knowledge, there is no clear evidence of prion protein toxicity in Purkinje cells. Only over-expression of the Doppel gene in Prnp-deficient mice has been correlated with Purkinje cell loss [59,60]. Our results support the possible protective function of Hsp70 in Purkinje cells and suggest a role of Hsp90 in this mechanism. Further analysis would be necessary to clarify the role of Hsp over-expression in these cells in prion diseases.

In CJD [58], the regions with severe spongiosis showed low immunoreactivity for Hsp72. In agreement with this report, we detected low expression of Hsp70 protein in one infected animal showing the greatest degree of spongiform alteration. In fact, the stepwise regression analysis revealed a negative association of Hsp70 protein with spongiosis in the cerebellum and diencephalon, suggesting that the reduction in the cytoprotective effect of Hsp70 could be involved in neuronal loss, at least in these two areas. However, the results of correlation and stepwise regression must be taken with caution due to the reduced amount of available data. Nevertheless, they illustrate the joint effect of several lesions of the gene or protein expression on the tissues analysed and pointed out the complexity of causes of modifications of gene and protein expression.

In conclusion, Hsp seem to be involved in gliosis and inflammatory reactions rather than in anti-apoptotic processes in natural scrapie. However, the intense immunoreactivity of Purkinje cells for Hsp70 and Hsp90, as well as the negative association of Hsp70 with prion protein immunolabelling and spongiosis, suggests a neuroprotective effect in this cell type against the stress related to ovine encephalopathy.

## Competing interests

The authors declare that they have no competing interests.

## Authors' contributions

CS carried out the gene and protein expression studies and wrote the manuscript. RB conceived and carried out the histopathological and immunohistochemical analysis. JL, AM-C, JHC and PZ participated in gene expression analysis and the interpretation of data. LV performed the statistical association studies. MS participated in the statistical analysis, interpreted the data and critically revised the manuscript. HF, CA and JJB participated in the histopathological analysis and evaluation. IM-B conceived of the study, participated in its design and coordination and wrote the paper. All authors discussed the results and implications, read and approved the final manuscript.

## Supplementary Material

Additional file 1 **Individual scoring for scrapie lesions**. Individual scoring for prion deposition, spongiosis, neuronal vacuolization, GFAP immunostaining, LGS staining and activated caspase-3 immunoreactivity for control (C) and scrapie (Sc)-infected sheep in the different areas analysed. Click here for file
