# Supplementary material for: Changes in HSP gene and protein expression in natural scrapie with brain damage
Source: Vet Res. 2011 Jan 24;42(1):13. doi: 10.1186/1297-9716-42-13 (PMC3037893; doi:10.1186/1297-9716-42-13)
Supplement: Additional file 1 — Individual scoring for scrapie lesions. Individual scoring for prion deposition, spongiosis, neuronal vacuolization, GFAP immunostaining, LGS staining and activated caspase-3 immunoreactivity for control (C) and scrapie (Sc)-infected sheep in the different areas analysed. [file 1297-9716-42-13-S1.DOC]

**Additional file 1:** Individual scoring for prion deposition, spongiosis, neuronal vacuolization, GFAP immunostaining, LGS staining and activated caspase-3 immunoreactivity for control (C) and scrapie (Sc)-infected sheep in the different areas analysed.

|  |  | Scrapie lesions | | | | | |
| --- | --- | --- | --- | --- | --- | --- | --- |
| Animals | Area | Prion | Spongiosis | Vacuolization | GFAP | LGS | Casp-3 |
| C1 | Cerebellum | 0 | 1 | 0 | 3 | 2 | 0 |
| C2 | Cerebellum | 0 | 1 | 0 | 3 | 2 | 0 |
| C3 | Cerebellum | 0 | 1 | 0 |  | 2 |  |
| C4 | Cerebellum | 0 | 2 | 0 | 3 | 2 | 1 |
| C5 | Cerebellum | 0 | 1 | 0 |  | 2 | 1 |
| Sc1 | Cerebellum | 4 | 4 | 0 | 5 | 3 | 0 |
| Sc2 | Cerebellum | 1 | 2 | 0 | 3 | 3 | 0 |
| Sc3 | Cerebellum | 2 | 1 | 0 |  | 4 | 1 |
| Sc4 | Cerebellum | 2 | 1 | 0 | 4 | 3 | 0 |
| Sc5 | Cerebellum | 5 | 4 | 0 | 5 | 3 | 2 |
| Sc6 | Cerebellum | 1 | 1 | 0 | 3 | 4 | 1 |
| Sc7 | Cerebellum | 3 | 1 | 0 | 5 | 3 | 0 |
| Sc8 | Cerebellum | 2 | 1 | 0 | 4 | 3 | 1 |
| C1 | Prefrontal Cortex | 0 | 0 | 0 | 2 | 1 | 1 |
| C2 | Prefrontal Cortex | 0 | 1 | 0 | 2 | 1 | 0 |
| C3 | Prefrontal Cortex | 0 | 0 | 0 | 3 | 1 |  |
| C4 | Prefrontal Cortex | 0 | 0 | 0 | 2 | 1 | 1 |
| C5 | Prefrontal Cortex | 0 | 0 | 0 | 3 | 1 | 1 |
| Sc1 | Prefrontal Cortex | 1 | 3 | 0 | 4 | 5 | 1 |
| Sc2 | Prefrontal Cortex | 1 | 2 | 1 | 3 | 4 | 0 |
| Sc3 | Prefrontal Cortex | 2 | 2 | 0 | 3 | 3 | 1 |
| Sc4 | Prefrontal Cortex | 3 | 2 | 0 | 3 | 3 | 0 |
| Sc5 | Prefrontal Cortex | 4 | 3 | 2 | 5 | 3 | 1 |
| Sc6 | Prefrontal Cortex | 2 | 1 | 0 | 3 | 3 | 0 |
| Sc7 | Prefrontal Cortex | 1 | 1 | 0 | 4 | 3 | 0 |
| Sc8 | Prefrontal Cortex | 1 | 1 | 0 | 4 | 5 | 2 |
| C1 | Diencephalon | 0 | 1 | 0 | 2 | 1 | 1 |
| C2 | Diencephalon | 0 | 1 | 0 | 2 | 2 | 1 |
| C3 | Diencephalon | 0 | 1 | 0 | 2 | 2 |  |
| C4 | Diencephalon | 0 | 1 | 0 | 3 | 2 | 0 |
| C5 | Diencephalon | 0 | 1 | 0 | 3 | 2 | 0 |
| Sc1 | Diencephalon | 5 | 4 | 1 | 5 | 4 | 2 |
| Sc2 | Diencephalon | 3 | 3 | 3 | 4 | 4 | 2 |
| Sc3 | Diencephalon | 1 | 2 | 3 | 3 | 4 | 0 |
| Sc4 | Diencephalon | 2 | 2 | 1 | 3 | 5 | 1 |
| Sc5 | Diencephalon | 4 | 4 | 1 | 5 | 2 | 0 |
| Sc6 | Diencephalon | 2 | 1 | 0 | 3 | 4 | 1 |
| Sc7 | Diencephalon | 2 | 2 | 0 | 5 | 4 | 0 |
| Sc8 | Diencephalon | 2 | 2 | 1 | 4 | 3 | 2 |
| C1 | Medulla oblon. | 0 | 1 | 0 | 3 |  | 1 |
| C2 | Medulla oblon. | 0 | 0 | 0 | 4 | 1 | 0 |
| C3 | Medulla oblon. | 0 | 0 | 0 | 3 | 1 |  |
| C4 | Medulla oblon. | 0 | 0 | 0 |  | 0 | 1 |
| C5 | Medulla oblon. | 0 | 1 | 0 | 3 | 0 | 1 |
| Sc1 | Medulla oblon. | 4 | 1 | 2 | 4 | 3 | 2 |
| Sc2 | Medulla oblon. | 2 | 1 | 0 | 4 | 4 | 2 |
| Sc4 | Medulla oblon. | 3 | 3 | 0 | 5 | 3 | 2 |
| Sc5 | Medulla oblon. | 4 | 3 | 2 | 5 | 4 | 0 |
| Sc6 | Medulla oblon. | 2 | 2 | 3 | 4 | 4 | 2 |
| Sc7 | Medulla oblon. | 3 | 2 | 0 | 5 | 4 | 1 |
| Sc8 | Medulla oblon. | 3 | 2 | 0 | 5 | 5 |  |
